# A Study on the Diagnosis of Lumbar Spinal Malposition in Chuna Manual Therapy Using X-Ray Images Based on Digital Markers

**DOI:** 10.3390/diagnostics15141748

**Published:** 2025-07-10

**Authors:** Min-Su Ju, Tae-Yong Park, Minho Choi, Younseok Ko, Young Cheol Na, Yeong Ha Jeong, Jun-Su Jang, Jin-Hyun Lee

**Affiliations:** 1 Department of Rehabilitation Medicine of Korean Medicine, Woosuk University Korean Medicine Hospital, 46 Eoeun-ro, Wansan-gu, Jeonju-si 54987, Republic of Korea; 2 Institute for Integrative Medicine, Catholic Kwandong University International St. Mary’s Hospital, 25 Simgok-ro 100 beon-gil, Seo-gu, Incheon 22711, Republic of Korea; 3 Digital Health Research Division, Korea Institute of Oriental Medicine, 1672 Yuseong-daero, Yuseong-gu, Daejeon 34054, Republic of Korea; 4 Department of Neurosurgery, Catholic Kwandong University International St. Mary’s Hospital, Catholic Kwandong University College of Medicine, 25 Simgok-ro 100 beon-gil, Seo-gu, Incheon 22711, Republic of Korea

**Keywords:** digital marker, Chuna manual therapy, X-ray, spinal malposition, diagnosis

## Abstract

**Background/Objectives:** This study aimed to evaluate digital markers and establish quantitative diagnostic criteria for spinal malpositions in Chuna manual therapy using lumbar X-rays. **Methods:** A total of 2000 X-ray images were collected from adult patients at the International St. Mary’s Hospital of Catholic Kwandong University. Five Chuna manual medicine experts annotated anatomical landmarks using a digital marker labeling program and diagnosed three types of spinal malpositions: flexion/extension, lateral bending, and rotation. Diagnostic accuracy was evaluated using weighted F1 (F1_W) scores, and the optimal threshold values for each malposition type were determined based on maximum F1_W performance. **Results:** The results showed high diagnostic performance, with average maximum F1_W scores of 0.76 for flexion/extension, 0.85 for lateral bending, and 0.71 for rotation. Based on this analysis, threshold angles for each type of spinal malposition in Chuna manual diagnosis were determined. **Conclusions:** This study demonstrates the diagnostic validity of digital marker-based X-ray analysis in Chuna manual therapy and is the first to propose quantitative diagnostic thresholds for spinal malpositions. These findings may serve as a foundation for clinical application in spinal assessment and treatment planning, with further validation studies warranted.

## 1. Introduction

Chuna manual therapy (CMT) is a traditional Korean manual treatment modality used by licensed Korean medicine doctors (KMDs) for diagnosing and treating structural and functional disorders of the musculoskeletal system using their hands, body parts, or assistive devices. Numerous studies have reported the therapeutic efficacy of CMT for spinal conditions such as herniated intervertebral disks, scoliosis, and spinal stenosis [1,2,3,4,5]. As with all medical procedures, the accurate assessment of the pathological state is essential in CMT. A critical aspect of CMT is the precise evaluation of joint malposition, and an independent diagnostic and therapeutic system has been developed to address this based on relative positional differences between vertebral segments [1].

Currently, diagnosis in CMT relies primarily on manual palpation. However, prior research has indicated that palpation has low reliability and validity, with inconsistent outcomes across repeated measures [6,7,8]. In clinical practice, palpation is commonly used to assess spinal alignment or malposition, but these methods are inherently subjective and heavily dependent on the examiner’s skill and experience [9]. Therefore, developing imaging-based diagnostic systems that minimize inter-examiner variability is essential to improve diagnostic accuracy and standardization in Chuna medicine. Particularly, a system that quantitatively assesses spinal alignment and dysfunction would enable the objective evaluation of treatment outcomes and, thus, improve the efficacy of the intervention [10]. Nevertheless, research and development focusing on imaging modalities specialized for CMT diagnosis remain limited.

As a supplementary approach to conventional palpation-based diagnosis, several studies have explored the use of plain radiography (X-ray) to assess spinal malposition. For example, Lee et al. [11] analyzed positional differences in spinal structures using X-ray images, but reported limitations in objectivity due to the absence of quantitative criteria for malposition. Subsequently, Lee et al. [12] visualized anatomical landmarks through consensus among CMT experts and proposed a diagnostic method using digital markers that can be used to analyze spinal malposition. A later study confirmed the inter-rater reliability of vertebral landmark labeling using this digital marker system [13]. The method showed high diagnostic reproducibility and inter-rater agreement within expert groups [9], demonstrating the potential of device-assisted diagnostics. Although several studies have utilized imaging modalities such as ultrasound and magnetic resonance imaging (MRI) to evaluate spinal alignment [14,15], standardized analytic protocols specific to CMT diagnosis are still lacking.

Despite ongoing research efforts, the development of clear quantitative diagnostic criteria for spinal malposition has remained a challenge. In the initial design phase of this study, we specifically reviewed preliminary data from two preceding studies by Lee [9] and Jang [13]. These prior investigations indicated a relatively high level of diagnostic consistency among Chuna medical experts in their assessments. However, despite this qualitative agreement, a critical gap persisted: the specific, quantitatively defined criteria underpinning these expert judgments were not clearly elucidated. This pivotal observation—that qualitative diagnostic consistency existed but lacked explicit quantitative underpinning—directly motivated the planning of the current research. Consequently, there remains a paucity of clearly defined quantitative criteria for determining spinal malposition or reference points for objectively analyzing relative vertebral positions. Establishing such criteria would support more rapid and consistent clinical decision-making and contribute to the standardization of education and research in this field. Therefore, this study aimed to evaluate the utility of digital markers by analyzing spinal malposition angles and diagnostic data from X-ray images and to establish quantitative diagnostic criteria for use in CMT.

## 2. Materials and Methods

### 2.1. Ethics and Trial Registration

This study was approved by the Institutional Review Board of the International St. Mary’s Hospital, Catholic Kwandong University (IRB No. 23 YEON IRB047). The trial was also registered with the Clinical Research Information Service (CRIS), operated by the Korea Disease Control and Prevention Agency (Trial registration number: KCT0008916; https://cris.nih.go.kr/cris/search/detailSearch.do?search_lang=E&focus=reset_12&search_page=L&pageSize=10&page=undefined&seq=25830&status=5&seq_group=25830, accessed on 2 February 2025).

### 2.2. Study Participants and Collection of Radiographic Cases

Anterior–posterior (AP) and lateral view lumbar spine X-ray images were collected from the radiographic database of International St. Mary’s Hospital, Catholic Kwandong University. All images were anonymized and converted to a Digital Imaging and Communications in Medicine (DICOM) format for research purposes.

### 2.3. Sample Size Calculation

As this is the first study to establish quantitative criteria for the diagnosis of malposition through Chuna, no directly comparable prior studies were available to guide sample size estimation. Based on the sample size formula (Equation (1)) with a 99% confidence level and a 3% margin of error, the minimum required sample size was calculated to be 1844 cases. Ultimately, a total of 2000 cases were selected for analysis.

(1) $$n=\frac{Z^{2}\cdot p\cdot (1-p)}{E^{2}}$$

*Z* denotes the critical value for a 99% confidence level (2.576); p represents the assumed population proportion (0.5, to ensure the maximum required sample size); and E indicates the desired margin of error (0.03).

### 2.4. Eligibility Criteria

#### 2.4.1. Inclusion Criteria

The inclusion criteria includes the following:

(a) Adult men and women aged 18–80 years;
(b) Patients who underwent lumbar spine plain radiography at International St. Mary’s Hospital, Catholic Kwandong University, either as part of a clinical visit or during follow-up in the Department of Neurosurgery.

#### 2.4.2. Exclusion Criteria

The exclusion criteria includes the following:

(a) History of spinal surgery involving instrumentation, such as spinal fusion or screw fixation;
(b) Presence of structural spinal abnormalities that preclude digital marker generation (e.g., tumors, fractures, lumbar sacralization, sacral lumbarization, or severe scoliosis);
(c) Individuals deemed unsuitable for participation by the research investigator.

### 2.5. CMT Spinal Diagnostic Panel

Chuna-based spinal malposition diagnoses using X-ray images were conducted by a panel of five evaluators. All evaluators were KMDs with over 5 years of clinical experience performing CMT and met at least one of the following qualifications: Korean rehabilitation medicine specialist, Korean Society of Chuna Manual Medicine for Spine and Nerves (KSCMM) standard curriculum instructors, members of the board of education who had completed KSCMM’s regular workshop courses, or those who had published a paper related to Chuna medical imaging of the spine.

### 2.6. Radiographic Analysis

A total of 2000 anonymized lumbar spine X-ray cases in the DICOM format were evenly distributed among the five evaluators. Each evaluator received training on a standardized operating procedure (SOP) for the diagnosis of malposition [11] and digital marker generation and precautions [11].

**Figure 1:**
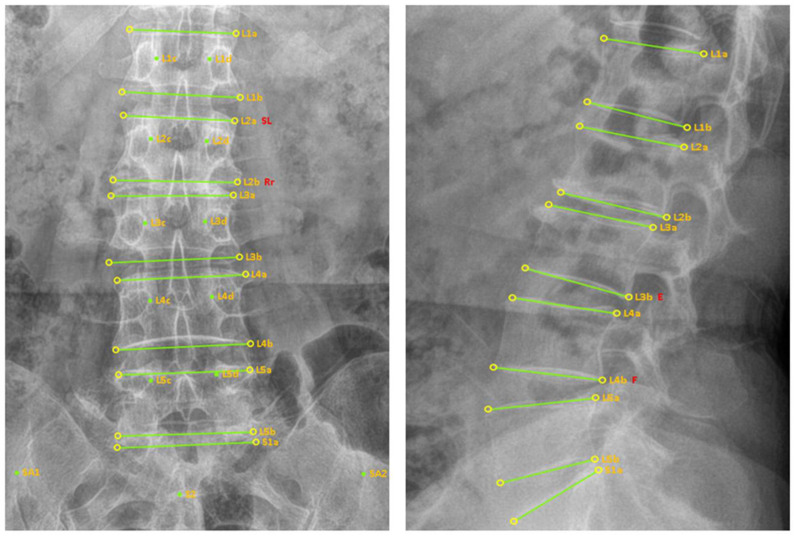
Examples of digital markers and malposition diagnostic labels.

The evaluators generated digital markers using a digital marker labeling program (Dicomlabel Ver. 3.0, Korea Institute of Oriental Medicine, Daejeon, Korea) based on the anonymized L-spine imaging data. They generated digital markers from the DICOM X-ray files per the SOP and identified malposition types (flexion/extension, lateral bending, and rotation) (Figure 1).

### 2.7. Digital Marker Generation Method

#### 2.7.1. AP View

**Figure 2:**
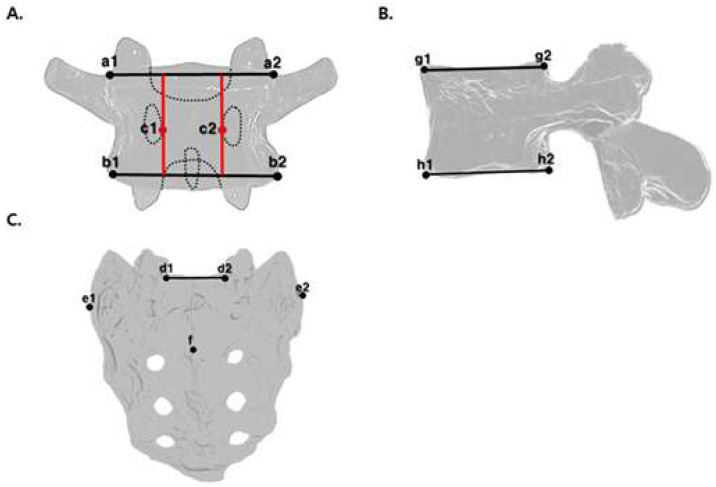
Digital marker generation locations: (**A**) lumbar spine in anteroposterior view; (**B**) lumbar spine in lateral view; (**C**) sacrum in anteroposterior view. Red lines in (**A**): Bilateral medial pedicle lines.

For each lumbar vertebra, a superior line was drawn by connecting the left and right endpoints of the superior endplate (a1, a2), and an inferior line was drawn using the corresponding endpoints of the inferior endplate (b1, b2). Additional markers were placed on the most medial points of the bilateral pedicles (c1, c2) (Figure 2A).

To diagnose malposition at L5, the following digital markers were generated for S1: endpoints of the superior surface (d1, d2), a line connecting the endpoints, the most lateral points of the sacral ala (e1, e2), and the midpoint of the spinous process of S2 (f) (Figure 2C).

#### 2.7.2. Lateral View

For each lumbar vertebra, a superior line was drawn by connecting the anterior and posterior endpoints of the superior endplate (g1, g2), and an inferior line was drawn by connecting the corresponding endpoints of the inferior endplate (h1, h2) (Figure 2B). Reference points on the first sacral vertebra (S1) are required for the diagnosis of malposition at L5. Two endpoints on the anterior and posterior surfaces of the S1 vertebral body and one line connecting them were used to generate the digital markers.

### 2.8. Method for Malposition Angle Analysis Based on Digital Markers

Spinal malposition angles were analyzed using digital markers with reference to previous studies [12,16,17].

#### 2.8.1. Flexion/Extension Angle

**Figure 3:**
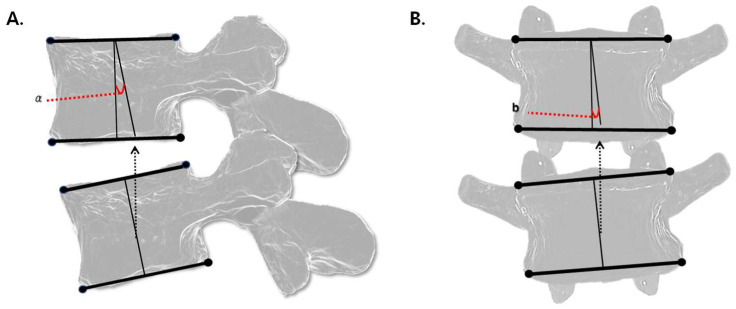
Methods for measuring malposition angles using digital markers, covering (**A**) flexion/extension and (**B**) lateral bending. a in (**A**): Flexion/extension angle; b in (**B**): Lateral bending angle.

Flexion and extension malpositions were evaluated using digital markers on lateral view X-ray images. The malposition angle (a) was calculated by measuring the angular difference between the centerlines of the superior and inferior vertebral bodies. Each centerline was defined by connecting the midpoints of the superior and inferior endplates of each vertebra (Figure 3A).

#### 2.8.2. Lateral Bending Angle

Lateral bending malpositions were assessed using digital markers on AP view images. The lateral bending angle was determined using the same method as for flexion/extension by comparing the centerlines of the adjacent vertebral bodies (Figure 3B). Directionality (left vs. right) was not distinguished in the analysis.

#### 2.8.3. Rotation Angle

**Figure 4:**
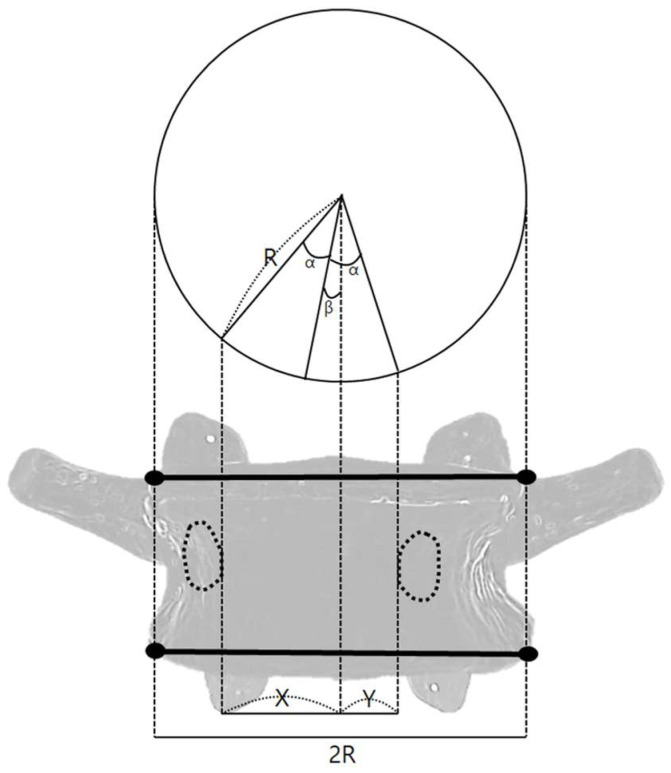
Methods for measuring malposition angles using digital markers, covering rotation.

Rotational malpositions were analyzed using digital markers on AP view X-ray images. The angle was calculated based on the angle between the vertebral centerline and the most medial points of the left and right pedicles (Equation (2)) [17]. Assuming the vertebral body is cylindrical, rotation angles were calculated by defining the horizontal distance from the vertebral midline to the inner edge of the right pedicle as X, the distance from the vertebral midline to the inner edge of the left pedicle as Y, the transverse diameter of the vertebral body as R, the angle between the inner pedicle points as 2α, and the rotational angle of the vertebra as β (Figure 4). These parameters were used to compute the individual vertebral rotation angle. Directionality (left vs. right) was not distinguished in the analysis.

$$\frac{X}{R}=sin\left(\alpha +\beta \right),\frac{Y}{R}=sin\left(\alpha -\beta \right)$$

(2) $$\alpha +\beta =\theta_{1},\alpha -\beta =\theta_{2}$$

$$∴\beta =\frac{{(\theta }_{1}-\theta_{2})}{2}$$

**Figure 5:**
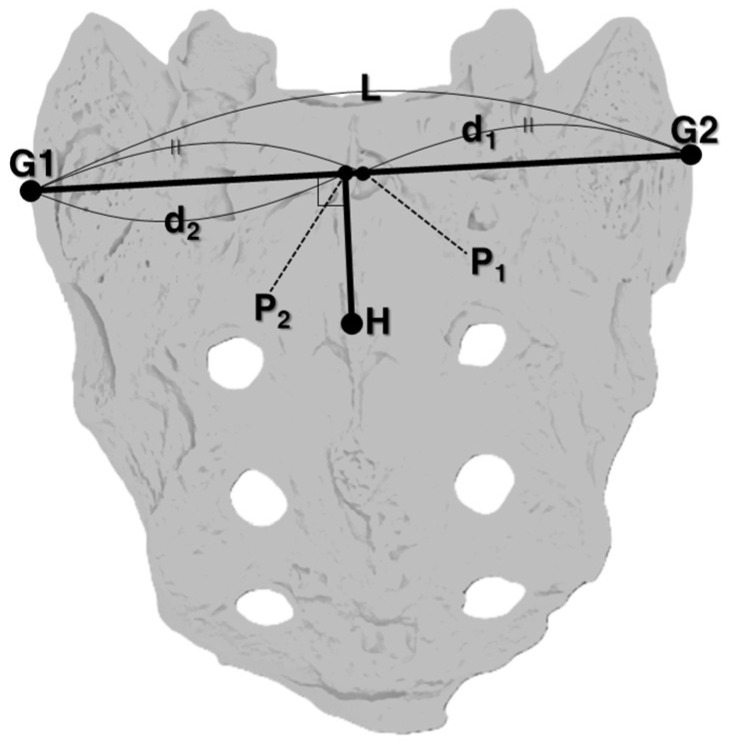
Assessment of sacral rotation.

An analysis of the rotational malposition at L5 was limited due to the lack of a standardized method for measuring sacral (S1) rotation. Therefore, rotational angle measurements for L5 were only included in cases where no rotation was identified at S1 [1]. The absence of any rotational malposition in S1 was determined as follows: A straight line L was drawn by connecting points G1 and G2. The midpoint of L was designated as P1, and the distance from G1 to P1 as d1. The point where a perpendicular from point H intersects L was designated P2. The distance from P2 to the nearer of either G1 or G2 was defined as d2. With r defined as $\frac{┃d1-d2┃}{d1}$, an r value closer to zero was interpreted as indicating no rotational malposition of the sacrum (Figure 5).

**Figure 6:**
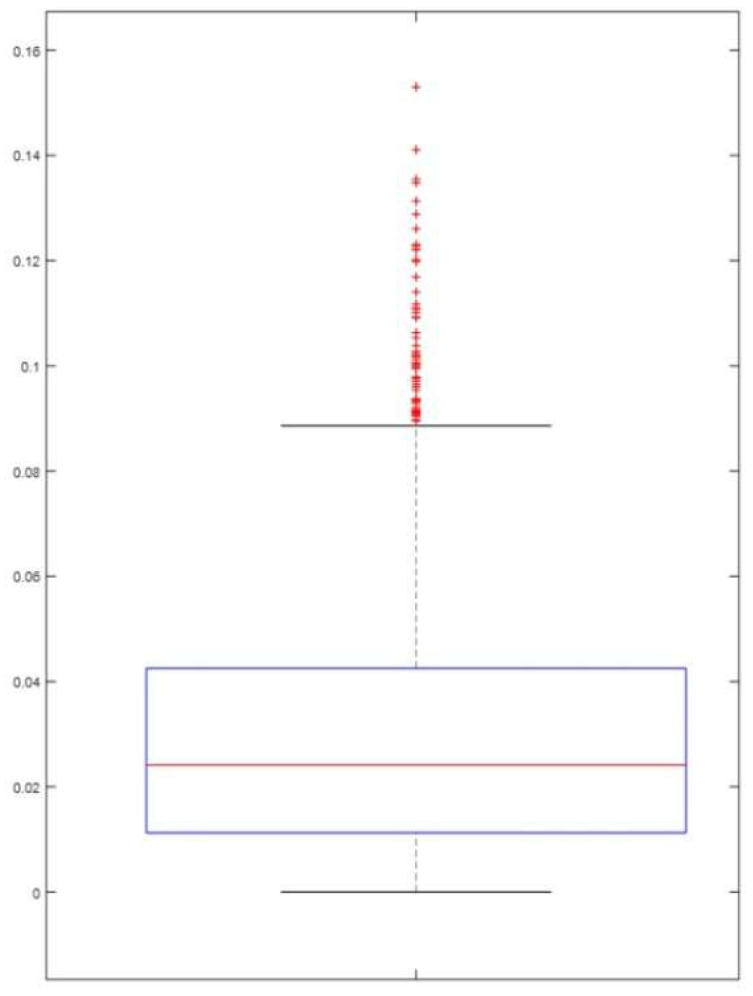
Distribution of r values.

The distribution of r values was visualized using a box plot. Samples below the outliers, defined as the third quartile (Q3) + 1.5 × interquartile range, were classified as having no sacral rotation. Figure 6 presents the distribution of r values used to determine the rotation of S1. The threshold for outliers was determined to be 0.0886. Only samples with r values below this threshold were considered as having no rotational malposition of S1, and only these samples were used for calculating the rotational angle of L5 as an isolated vertebral segment.

### 2.9. Result Derivation Method

The digital markers generated by the evaluators were analyzed to calculate vertebral malposition angles for each segment. All angle calculations were performed using Python (ver. 3.9.12, Python Software Foundation, Wilmington, DE, USA). The accuracy of each evaluator’s diagnosis was assessed based on a predefined malposition angle threshold. The threshold value that produced the highest mean diagnostic accuracy across evaluators was selected as the final cutoff value for malposition classification.

### 2.10. Statistical Analysis

Statistical methods were referenced from previous studies analyzing lumbar spine images [18]. The results of vertebral malposition angle calculations and evaluator diagnoses were organized into confusion matrices. Diagnostic accuracy was evaluated by calculating the weighted F1 score (F1_W score) based on these confusion matrices. Two types of thresholding were used to assess diagnostic performance: the global threshold, which represents a single optimal cutoff value applied uniformly across all raters to establish objective diagnostic criteria, and the local threshold, which was individually optimized for each rater to assess intra-rater consistency. An F1_W score of ≥0.7 indicated a meaningful level of diagnostic accuracy [19]. All confusion matrix generation and F1_W score calculations were performed using MATLAB (ver. R2023a, MathWorks, Seoul, Republic of Korea).

## 3. Results

### 3.1. Sample Selection

**Figure 7:**
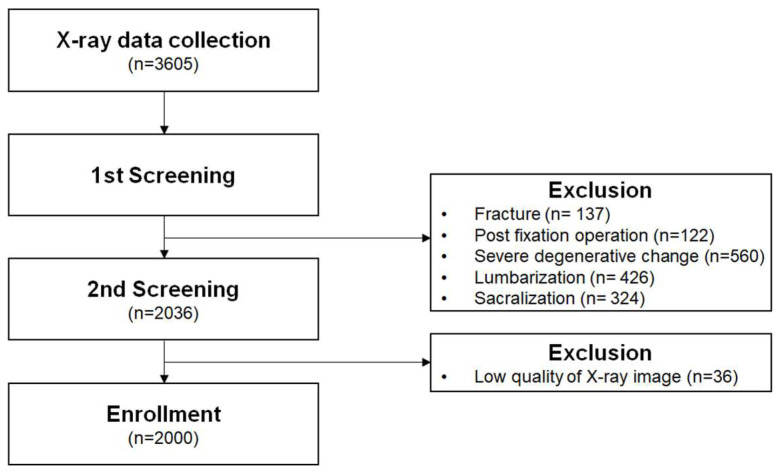
Study flowchart of patient selection.

A total of 3605 lumbar spine (L-spine) X-ray cases were retrieved from the Department of Neurosurgery database at International St. Mary’s Hospital, Catholic Kwandong University, between 1 January 2019, and 31 December 2022. Based on the exclusion criteria, 137 cases involving fractures, 122 cases with metal instrumentation, 560 cases with severe degenerative changes, 426 cases of sacralization of the lumbar spine, and 324 cases of lumbarization of the sacrum were excluded. Additionally, 36 cases with poor image quality were removed. A final sample of 2000 X-ray cases was included in the analysis (Figure 7).

### 3.2. Number of Malpositioned Vertebral Bodies

A total of 2625 vertebrae were diagnosed with flexion malposition and 455 vertebrae with extension malposition, indicating that flexion malposition was approximately 5.8 times more common than extension malposition. Regarding lateral bending, 1292 vertebrae were diagnosed with left bending malposition and 1075 vertebrae with right bending malposition, showing a higher frequency of left-sided malpositions. Since S1 rotation angles could not be measured, rotational malposition analysis was limited to L1–L4 and not L5. Among these, left rotation (1138 vertebrae) was approximately 1.9 times more common than right rotation (607 vertebrae) (Table 1).

### 3.3. Malposition Diagnostic Accuracy

#### 3.3.1. Flexion/Extension Malposition

Both the global threshold F1_W score (0.76) and the local threshold F1_W score (0.84) were statistically significant (Table 2).

#### 3.3.2. Lateral Bending Malposition

Both the global threshold F1_W score (0.85) and the local threshold F1_W score (0.87) were statistically significant (Table 2).

#### 3.3.3. Rotation Malposition

Both the global threshold F1_W score (0.71) and the local threshold F1_W score (0.72) were statistically significant (Table 2).

### 3.4. Optimal Angles for Maximum Diagnostic Accuracy

The threshold values yielding the highest scores were identified in the analysis of diagnostic accuracy (F1_W) for each type of malposition, and the reference angles for CMT-based malposition diagnosis were proposed based on these values (Table 3).

## 4. Discussion

The diagnostic framework for malposition in Chuna manual medicine has evolved to assess both structural abnormalities and functional impairments of the spine. With early models, the Palmer-Gonstead and National Diversified classification systems were introduced to define and diagnose spinal malposition [20]. Later, updated classification systems based on standard kinesiologic terminology and the Medicare nomenclature refined the categories into flexion, extension, lateral bending, and rotation [20].

Traditionally, palpation has been the primary method used to apply these diagnostic frameworks in clinical practice. However, previous studies have reported low reliability for palpation-based diagnosis [6,7,21], highlighting the need for more objective and quantitative methods. In this context, research on X-ray image analysis using digital markers has emerged [12]. Building on this trend, this study evaluated the diagnostic utility of digital markers and proposed quantifiable reference angles applicable to malposition diagnosis in CMT.

The key findings of this study are as follows: First, flexion malpositions were approximately 5.8 times more prevalent than extension malpositions, which can be attributed to spinal biomechanics and modern lifestyle factors. During lumbar flexion, posterior structures—particularly the facet joints—are separate, and the posterior ligaments are tensioned, resulting in reduced bony stability [22]. Furthermore, prolonged sitting or repetitive flexion activities can lead to the adaptive shortening of anterior structures and laxity of posterior ligaments, contributing to a resting flexed alignment [23]. This type of lumbar segmental instability is reportedly the most common clinical pattern [23]. Such instability is often associated with the loss of segmental lumbar lordosis and a flexion fixation pattern, which may have been objectively reflected by the quantitative imaging analysis in our study. Second, the diagnostic threshold for flexion/extension malposition tended to increase in the lower lumbar vertebrae. This likely reflects the natural anatomical curvature of the lumbar spine, which becomes more pronounced in the lower segments [24,25], which has likely guided experts to set higher angular thresholds for malposition at these levels. Third, the diagnostic accuracy for rotational malposition was lower than that for flexion/extension and lateral bending. This may be multifactorial, with factors such as evaluators’ consideration of spinous process orientation and asymmetry in pedicle size having some effect [11]. The degeneration of the spinous processes or pedicles may introduce diagnostic ambiguity. For example, degenerative changes in the spinous processes that lead to increased height or irregular morphology [26] or irregularity may provide inaccurate rotation data. Similarly, left–right differences in pedicle size [27] may contribute to diagnostic errors.

The strengths of this study are as follows: First, this is the largest-scale study to date on malposition diagnosis in Chuna medicine (sample of 2000 X-ray cases). The large sample size helped overcome the limitations of small-scale studies, such as random variation, increased error, bias, and reduced accuracy [28]. Second, an SOP was established based on Chuna textbooks and the prior literature, and all evaluators were trained accordingly. This ensured consistent digital marker generation and malposition diagnosis across evaluators. Third, while various diagnostic studies have been conducted in complementary and manual medicine [29,30,31], few have quantitatively defined diagnostic criteria based on the characteristics of the intervention. This study is the first to propose quantitative reference standards for spinal malposition specific to CMT. Fourth, the average F1_W scores for all malposition types exceeded 0.7, confirming statistically significant diagnostic performance. Particularly, high diagnostic accuracy was consistently observed in both global thresholds, reflecting the level of agreement among different raters (inter-rater reliability), and local thresholds, which assess the consistency within individual raters (intra-rater reliability). These results support the robustness and reproducibility of the proposed quantitative diagnostic criteria both across and within raters [12]. Fifth, using digital markers, we quantitatively assessed segmental spinal malpositions by analyzing both L-spine AP and lateral X-ray images. This approach is distinct from the conventional Cobb angle method, which evaluates global spinal curvature across multiple segments using only lateral X-ray images [32]. This allows for a more detailed and localized diagnosis tailored to the specific treatment targets in CMT, thereby bringing a comparable level of objectivity and standardization to the nuanced diagnostic criteria used in CMT that have traditionally relied on subjective palpation.

However, several limitations should be noted. First, each rater analyzed different sets of radiographs, making it impossible to directly assess inter-rater reliability. Nevertheless, the high F1_W scores under global thresholds suggest acceptable diagnostic consistency. Future studies using identical datasets are needed to rigorously evaluate inter-rater agreement, especially regarding digital marker placement. Second, despite standardized SOP training for digital marker placement, substantial variability in malposition diagnoses was observed among raters, with one rater identifying 985 flexion malpositions and another only 52. This reflects the inherent subjectivity of CMT-based diagnosis, even with consistent landmark generation. These findings emphasize the need for objective, quantitative diagnostic thresholds to reduce interpretation bias. Third, although all raters followed a standardized SOP, the absence of an independent verification process to confirm full protocol compliance may have introduced measurement variability. Future research should implement automated marker placement methods to enhance precision. Such approaches could improve both diagnostic accuracy and reproducibility. Fourth, this study proposed quantitative thresholds for CMT-based X-ray malposition diagnosis but did not investigate associations between malpositions and clinical factors such as pain location, intensity, or related symptoms. This limits the direct clinical interpretation of the findings. Further research should explore these associations to integrate objective X-ray criteria into evidence-based CMT diagnosis and treatment. Finally, the current study focused only on the lumbar spine. The validation of these quantitative thresholds across other spinal regions where CMT is applied is necessary. Such research will help establish the generalizability of our proposed diagnostic criteria. Furthermore, to strengthen the validity and clinical applicability of our proposed diagnostic thresholds, future studies should independently verify inter-examiner agreement on both diagnosis and biomarker placement. Such research should involve examiners who did not participate in the initial threshold determination to enable more independent and robust validation.

Overall, this study provides meaningful groundwork for establishing objective criteria in Chuna-based malposition diagnosis using digital markers. Follow-up clinical research is needed to assess whether applying these diagnostic methods improves treatment outcomes. If validated, these criteria could support a more rational and effective application of CMT in clinical practice. Nevertheless, methodological refinements are necessary, particularly regarding the standardization of digital marker placement and validation of angle measurements. Such improvements are essential to minimize diagnostic errors and promote the more consistent and effective use of CMT in both research and clinical settings.

## 5. Conclusions

This study analyzed the CMT-based diagnosis of spinal malposition using X-ray imaging with digital markers. The highest average diagnostic accuracy scores (F1_W) achieved by the evaluators were 0.76 for flexion/extension malposition, 0.85 for lateral bending malposition, and 0.71 for rotational malposition, all of which were statistically significant. Additionally, threshold values yielding the highest diagnostic accuracy were identified for each type of malposition, thereby establishing quantitative reference criteria.

This study demonstrated the utility of digital marker-based X-ray analysis in the diagnosis of spinal malposition in Chuna manual medicine and established quantitative diagnostic thresholds. These reference values may serve as a practical tool for clinical application in both malposition assessment and treatment planning. Further research is needed to validate their clinical applicability and effectiveness in diverse practice settings.

## Figures and Tables

**Table 1:** Distribution of malposition diagnoses by diagnosticians.

	Flexion (*n*/2000)	Extension (*n*/2000)	Rt. Lateral Bending (*n*/2000)	Lt. Lateral Bending (*n*/2000)	Rt. Rotation (*n*/1600) *	Lt. Rotation (*n*/1600) *
Rater 1	985	44	320	453	170	171
Rater 2	451	102	147	195	60	253
Rater 3	945	131	358	365	202	391
Rater 4	52	34	188	216	101	210
Rater 5	192	144	62	63	74	113
Total	2625	455	1075	1292	607	1138

* Each rater conducted evaluations for 400 cases × five lumbar levels (*n* = 2000), excluding rotation assessment. However, for rotation assessment, since the rotation angle of the S1 level cannot be quantitatively evaluated, evaluations were conducted only for four lumbar levels, excluding the rotation angle of L5 (*n* = 1600).

**Table 2:** Diagnostic accuracy (F1_W scores) for spinal malposition types.

	F1_W Score					
	Global Threshold			Local Threshold		
	Flexion/Extension	Lateral Bending	Rotation	Flexion/Extension	Lateral Bending	Rotation
Rater 1	0.81	0.93	0.72	0.89	0.94	0.73
Rater 2	0.76	0.91	0.75	0.78	0.91	0.76
Rater 3	0.72	0.67	0.52	0.79	0.70	0.55
Rater 4	0.80	0.86	0.73	0.95	0.86	0.74
Rater 5	0.70	0.88	0.82	0.81	0.94	0.84
Average ±SD	0.76 ±0.04	0.85 ±0.09	0.71 ±0.10	0.84 ±0.07	0.87 ±0.09	0.72 ±0.10

**Table 3:** Optimal angles for maximum diagnostic accuracy by malposition type.

	Vertebral Body				
	L1	L2	L3	L4	L5
Flexion (°)	0.64	3.56	5.29	8.90	8.53
Extension (°)	10.46	14.95	17.61	20.76	24.47
Lateral bending (°)	2.21	2.10	1.92	2.06	2.31
Rotation (°)	9.49	5.19	4.59	5.87	7.13

## Data Availability

The data presented in this study are available upon reasonable request.
